# Systemic Lupus Erythematosus Flare Triggered by Mosquito Bite Manifesting as Febrile Targetoid Rash

**DOI:** 10.1002/ccr3.9714

**Published:** 2024-12-06

**Authors:** Joe Khodeir, Hamad El Hajj, Paul Ohanian

**Affiliations:** ^1^ Department of Dermatology, Faculty of Medicine and Medical Sciences University of Balamand, Saint Georges Hospital University Medical Center Beirut Lebanon; ^2^ Division of Dermatology, Saint George University of Beirut Saint Georges Hospital University Medical Center Beirut Lebanon; ^3^ Department of Family Medicine, Faculty of Medicine and Medical Sciences University of Balamand, Saint Georges Hospital University Medical Center Beirut Lebanon

**Keywords:** insect bite, lupus flare, systemic lupus erythematosus, targetoid rash

## Abstract

Insect bites can trigger disease flare in systemic lupus erythematosus, including atypical targetoid rash. Awareness of such triggers and appropriate preventive measures are crucial for effective SLE management.

## 1 Introduction

Systemic lupus erythematosus (SLE) is a chronic autoimmune disorder characterized by a wide range of clinical manifestations and periods of exacerbation and remission [1]. Cutaneous manifestations are frequently observed in SLE patients and can range from classic malar rash to more atypical presentations. Among these, the targetoid rash is less commonly associated with SLE and may pose diagnostic challenges [2, 3, 4]. This rash, characterized by a central erythematous area surrounded by a pale zone and a peripheral ring of erythema, can mimic other dermatological conditions [5, 6]. Various triggers are known to precipitate SLE flares, although some are less commonly recognized in the literature. These triggers may include ultraviolet light, infections, stress, and hormonal changes [7]. Insect bites, while noted as potential triggers in very rare cases, are not widely documented as a significant factor in SLE exacerbations [8].

This case report describes an unusual presentation of an SLE flare, marked by a targetoid rash and associated with recent mosquito bites. By presenting this rare clinical scenario, the report aims to enhance awareness of atypical SLE manifestations and underscores the importance of recognizing diverse triggers in the management of SLE. It also highlights the need for preventive measures, including insect protection, to mitigate the risk of flare‐ups in susceptible patients.

## 2 Case History/Examination

A 39‐year‐old female with a 2‐year history of SLE, managed with oral hydroxychloroquine (200 mg twice daily) and sun protection, presented with a rash on her upper chest, fever, and joint pain that had developed over the past 2 days. The rash was intensely pruritic and expanding. Additionally, she experienced bilateral joints pain on both wrists and knees, fatigue, and headache. She denied any sun exposure or recent illness but reported mosquito bites on the affected area on her chest.

**FIGURE 1:**
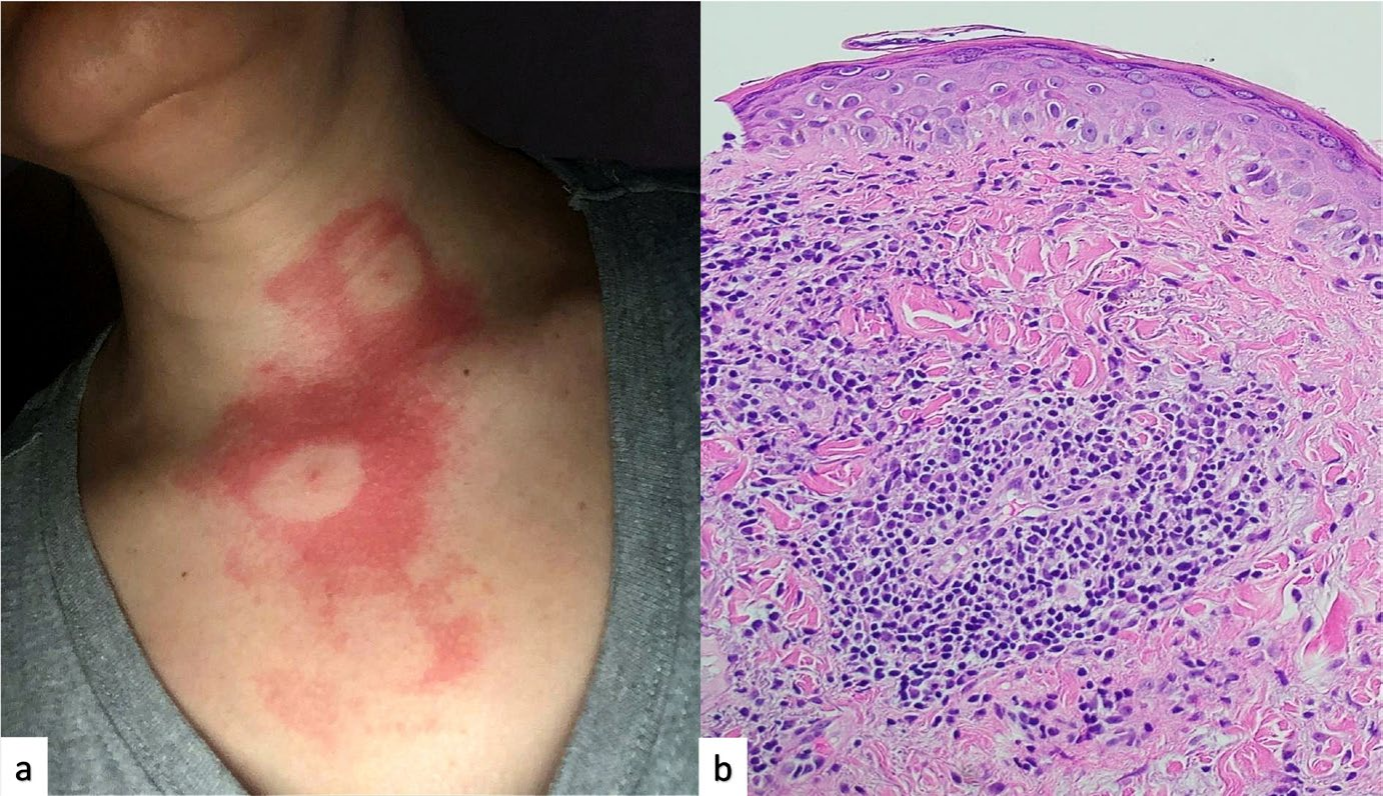
(a) SLE flare after mosquito bites manifesting as two annular targetoid lesions. (b) Skin biopsy showing basal cell layer vacuolar changes, civatte bodies, lymphocytic and histiocytic infiltrates in the dermis around blood vessels and hair follicles, consistent with cutaneous lupus erythematosus.

Examination revealed an erythematous macular rash on her upper chest, annular in appearance with a central red dot, surrounded by a clearing area and severe erythema, forming a bull's eye or target shape (Figure 1a). No lymphadenopathy was present, but joint tenderness was noted over both wrists and knees without any swelling.

## 3 Methods (Differential Diagnosis, Investigations, and Treatment)

Laboratory tests showed elevated antinuclear antibody levels (titer 1/450), high ESR (60 mm/h), low C3 and C4 levels (6 and 2 mg/dL respectively), and mild thrombocytopenia (120,000 μL), indicating a flare of SLE. Anti‐Ro/SSA, anti‐La/SSB, and rheumatoid factor were negative, ruling out Rowell Syndrome. Given the targetoid nature of the rash, an infectious etiology such as Lyme disease or acute bacterial infection was considered. However, the patient had no history of tick bites and did not live in an endemic area. Lyme disease serology, including IgM and IgG, was negative, as were blood cultures. Due to the atypical rash, a skin biopsy with direct immunofluorescence (DIF) was performed, revealing basal cell layer vacuolar changes, civatte bodies, and lymphocytic and histiocytic infiltrates in the dermis around blood vessels and hair follicles, consistent with cutaneous lupus erythematosus (Figure 1b). The patient was started on oral prednisone at 1 mg/kg for 1 week, followed by a tapering regimen over the next 2 weeks.

## 4 Results (Outcome and Follow‐Up)

Two days after starting the systemic steroids, the patient became afebrile, along with rapid amelioration in joints pain. Her targetoid rash started to regress and became less erythematous. This regimen led to rapid improvement in her symptoms with total remession 5 days later. Ten days after the initiation of the systemic steroids, hydroxychloroquine was increased to 200 mg three times daily for maintenance. One month later, the patient reported no relapse of symptoms and was off systemic steroids. Comprehensive sun protection and insect repellent were recommended to prevent future flares.

## 5 Discussion

SLE is a complex autoimmune disease characterized by the production of autoantibodies and chronic inflammation that can affect multiple organ systems, including the skin, joints, kidneys, and central nervous system [4]. The disease's heterogeneity presents significant diagnostic and management challenges [2]. Common triggers for SLE exacerbations include ultraviolet (UV) light exposure, infections, and psychological or physical stress, all of which can stimulate immune activity and potentially lead to disease flares. Additionally, medications and hormonal changes are recognized factors that may exacerbate SLE symptoms. However, the role of insect bites as potential triggers for SLE flares is less commonly documented in the literature [7].

This case report adds to the existing literature by highlighting a unique association between a mosquito bite and an SLE flare. Unlike more typical triggers, the mosquito bite in this patient precipitated an SLE flare characterized by an unusual targetoid rash. While insect bites are known to induce localized allergic reactions and sometimes systemic responses, their role in triggering SLE exacerbations remains an area of limited understanding.

Two prior reports have documented SLE exacerbations following insect bites, which provide valuable context for this case. Javadi Parvaneh et al. reported a 13‐year‐old girl who developed SLE after a mosquito bite on her left cheek, presenting with a malar rash, oral ulcers, photosensitivity, and positive serologic tests [8]. Martín Nares et al. described a 38‐year‐old woman who experienced a severe SLE flare following a spider bite, initially presenting with fever, cough, and localized skin ulceration, which progressed to systemic symptoms [9]. These cases underscore the potential for insect bites to act as a catalyst for SLE flares, although the presentations were more typical of SLE‐associated rashes, unlike the targetoid rash observed in our patient.

The atypical targetoid rash in this case is a rare manifestation in SLE [2, 3, 4]. This unique presentation emphasizes the need for clinicians to consider a broad differential diagnosis, including the potential for insect bites to trigger unusual cutaneous manifestations in SLE patients [7]. Preventive measures, such as effective insect protection and rigorous sun protection, are essential strategies in minimizing the risk of flares in susceptible patients.

## 6 Conclusion

This case highlights a rare presentation of an SLE flare triggered by mosquito bites, resulting in an atypical targetoid rash. It underscores the importance of considering environmental factors, such as insect bites, as potential triggers in SLE management. Clinicians should remain vigilant for unusual manifestations and educate patients on preventive measures to reduce the risk of future flares.

## Author Contributions

**Joe Khodeir:** conceptualization, methodology, validation, writing – original draft, writing – review and editing. **Hamad El Hajj:** conceptualization, investigation. **Paul Ohanian:** supervision, validation.

## Consent

Written informed consent was obtained from the patient to publish this report, in accordance with the journal's patient consent policy.

## Conflicts of Interest

The authors declare no conflicts of interest.

## Data Availability

The data used to support the findings of this study are included within the article.
